# Elevated Brain Fatty Acid Amide Hydrolase Induces Depressive-Like Phenotypes in Rodent Models: A Review

**DOI:** 10.3390/ijms22031047

**Published:** 2021-01-21

**Authors:** Dorsa Rafiei, Nathan J. Kolla

**Affiliations:** 1 Institute of Medical Science, University of Toronto, Toronto, ON M5S 1A8, Canada; dorsa.rafiei@camh.ca; 2 Centre for Addiction and Mental Health (CAMH), Toronto, ON M5T 1R8, Canada

**Keywords:** endocannabinoids, fatty acid amide hydrolase, major depressive disorder, animal models of depression

## Abstract

Altered activity of fatty acid amide hydrolase (FAAH), an enzyme of the endocannabinoid system, has been implicated in several neuropsychiatric disorders, including major depressive disorder (MDD). It is speculated that increased brain FAAH expression is correlated with increased depressive symptoms. The aim of this scoping review was to establish the role of FAAH expression in animal models of depression to determine the translational potential of targeting FAAH in clinical studies. A literature search employing multiple databases was performed; all original articles that assessed FAAH expression in animal models of depression were considered. Of the 216 articles that were screened for eligibility, 24 articles met inclusion criteria and were included in this review. Three key findings emerged: (1) FAAH expression is significantly increased in depressive-like phenotypes; (2) genetic knockout or pharmacological inhibition of FAAH effectively reduces depressive-like behavior, with a dose-dependent effect; and (3) differences in FAAH expression in depressive-like phenotypes were largely localized to animal prefrontal cortex, hippocampus and striatum. We conclude, based on the animal literature, that a positive relationship can be established between brain FAAH level and expression of depressive symptoms. In summary, we suggest that FAAH is a tractable target for developing novel pharmacotherapies for MDD.

## 1. Introduction

Major depressive disorder (MDD) is one of the leading causes of disability worldwide and one of the most prevalent psychiatric disorders, placing a large burden on society and healthcare costs [1]. The likelihood of relapse increases with each subsequent major depressive episode (MDE) [2]. Clinically, an MDE is characterized by symptoms, such as depressed mood, anhedonia, feelings of guilt or worthlessness, disruptions in cognitive function and self-harm or suicide [3]. It is widely acknowledged that MDD is a heterogeneous condition, and approximately 50% of individuals with MDD do not respond to first-line treatments, which primarily target monoamines [4,5]. This observation has turned the focus from the traditional monoamine hypothesis of depression to other neurochemical systems that may not be targeted by first-line, monoamine-elevating antidepressants, such as selective serotonin reuptake inhibitors (SSRIs) [6].

**Figure 1:**
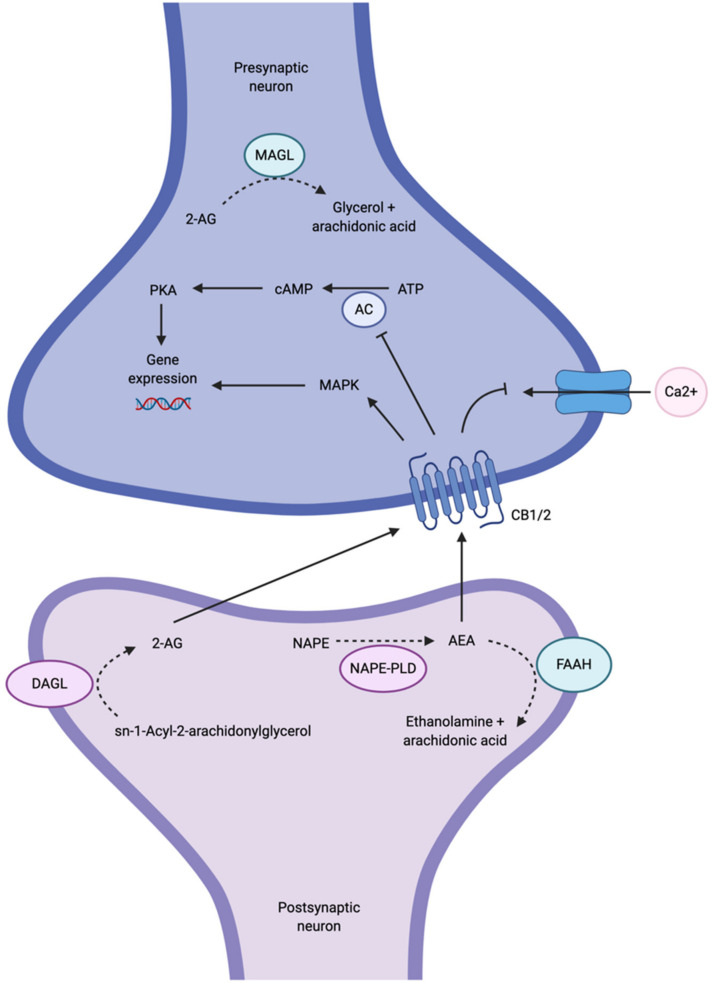
Major anandamide-CB1 receptor signaling pathways. Anandamide (AEA) is synthesized from N-arachidonoyl-phosphatidyl-ethanolamine (NAPE) by N-acylphosphatidylethanolamine-selective phospholipase D (NAPE-PLD) in the postsynaptic neuron. 2-arachidonylglycerol (2-AG) is also synthesized in the postsynaptic neuron from sn-1-Acyl-2-arachidonylglycerol by diacylglycerol lipase (DAGL). AEA and 2-AG are released into the synaptic cleft and bind CB1 and CB2 receptors, respectively, on the presynaptic axon terminal. Once CB1 and CB2 receptors are bound to their ligands, internal calcium currents are reduced. Adenylyl cyclase (AC) activity is also inhibited, subsequently inhibiting cyclic AMP (cAMP) and the protein kinase A (PKA) phosphorylation pathways. Alternatively, mitogen-activated protein kinase (MAPK) is activated. Both events lead to the regulation of gene expression. AEA is metabolized by fatty acid amide hydrolase (FAAH) to ethanolamine and arachidonic acid in the postsynaptic neuron. 2-AG is metabolized by monoacylglycerol lipase (MAGL) in the presynaptic neuron. Created with BioRender.com.

There is growing interest in the endocannabinoid system (ECS) as an alternative signaling pathway involved in MDD, since components of the ECS are highly expressed in fronto-limbic brain regions known to be affected in MDD, including the prefrontal cortex (PFC), hippocampus and amygdala [7]. Dysfunction in these regions has important functional implications in MDD. For example, dysfunction of the PFC affects many cognitive functions, such as decision making, attention, motivation and emotion regulation [8,9]. In the hippocampus, dysfunction may result in learning and memory impairments as well as compromised emotional and stress reactivity [9]. The amygdala is also known to be important for affective modulation and memory encoding [9]. Disruption of these key brain regions may result in functional impairment and emotional dysregulation in MDD. The ECS signaling pathway is composed of two main G protein-coupled receptors, cannabinoid 1 (CB1) and cannabinoid 2 (CB2) receptors, and two main endogenous cannabinoids (or endocannabinoids), N-arachidonylethanolamide (anandamide or AEA) and 2-arachidonylglycerol (2-AG), although other endocannabinoids are known to exist but are not as well-studied [10]. AEA and 2-AG are produced in the postsynaptic neuron on demand; that is, the endocannabinoids are not stored in vesicles, but rather they are biosynthesized and released as required [10]. AEA is synthesized from N-arachidonoyl-phosphatidyl-ethanolamine (NAPE) by N-acylphosphatidylethanolamine-selective phospholipase D (NAPE-PLD), whereas 2-AG is synthesized from sn-1-Acyl-2-arachidonylglycerol by diacylglycerol lipase (DAGL). NAPE-PLD and DAGL are stimulated by high calcium concentrations; therefore, calcium influx into the postsynaptic neuron is thought to trigger biosynthesis of AEA and 2-AG [10]. The endocannabinoids are then released into the synaptic cleft and bind, in a retrograde fashion, to CB1 and CB2 receptors that are located on axon terminals on the presynaptic neuron (Figure 1) [7]. The mechanism by which endocannabinoids are released into and taken up from the synaptic cleft, whether it is by passive diffusion across the plasma membrane or whether there is a membrane transporter involved, or both, is a matter of current debate (for a review of endocannabinoid transport, see Fowler [11]).

Although AEA and 2-AG are ligands for both CB1 and CB2 receptors, CB1 receptors are primarily targeted by AEA as well as Δ^9^-tetrahydrocannabinol (Δ^9^-THC), the psychoactive ingredient in marijuana [5,12]. Conversely, CB2 receptors are primarily targeted by 2-AG. Both CB1 and CB2 receptors are coupled to G_i/o_ proteins that, when stimulated, inhibit adenylyl cyclase (AC) activity, subsequently inhibiting cyclic AMP (cAMP) and the protein kinase A (PKA) phosphorylation pathways, or activating mitogen-activated protein kinase (MAPK) (Figure 1) [10]. Both events have the ability to regulate gene expression [10]. The activation of G_i/o_ proteins also reduces internal calcium currents; they are thus capable of regulating neurotransmitter release [4]. The net effect of ECS activity on synaptic transmission is dependent on whether CB1 receptors are found on GABAergic (inhibitory) or glutamatergic (excitatory) terminals [4]. To terminate endocannabinoid signaling, AEA is metabolized by fatty acid amide hydrolase (FAAH) in the postsynaptic neuron, and 2-AG is metabolized by monoacylglycerol lipase (MAGL) in the presynaptic neuron [7].

FAAH metabolizes AEA into ethanolamine and arachidonic acid, thereby terminating endocannabinoid signaling [13]. FAAH possesses both amidase and esterase activity, which allows the enzyme to hydrolyze *N*-acylethanolamines (e.g., oleamide) and monoacylglycerols (e.g., 2-AG), respectively [13]. It was first reported in 1993 that FAAH additionally hydrolyzes AEA [14]. FAAH belongs to a family of enzymes, named amidases, that hydrolyze fatty acid amide bonds [13]. FAAH is an integral membrane serine hydrolase composed of two subunits, characterized by a core structure of a twisted β-sheet surrounded by 24 α-helices [15]. FAAH possesses five key regions that are important for its enzymatic function: (1) the membrane-binding cap, which anchors the enzyme into the cytoplasmic leaflet of the lipid bilayer; (2) the membrane-access channel through which lipophilic substrates access the active site from the membrane; (3) the substrate-binding pocket, where the acyl chain of the substrate resides during catalysis; (4) the active site, which contains the highly conserved Ser241-Ser217-Lys142 catalytic triad; and (5) the cytosolic access channel, which serves as an exit path for the polar head group of the substrate after hydrolysis [16]. Covalent inhibitors of FAAH bind at Ser241, which blocks the catalytic function of FAAH [17].

Mouse and human *FAAH* genes are located on chromosomes 4 and 1, respectively, and share 84% amino acid sequence homology [18]. A single-nucleotide polymorphism exists in the *FAAH* gene (rs324420, C385A), whereby threonine replaces proline at position 129. This mutation makes the protein more susceptible to proteolysis, resulting in reduced activity [19]. The wild-type genotype, C/C, is associated with higher FAAH expression and protein activity, whereas the variant genotype, C/A or A/A, is associated with lower FAAH expression and protein activity [20,21]. For example, individuals possessing the A/A genotype exhibit reduced stress reactivity [22,23]. Recent studies have suggested that the ECS undergoes epigenetic modulation by diet, stress, drugs, alcohol, smoking or exercise [24]. For example, reduced DNA methylation at the *FAAH* gene was found in a study of late-onset Alzheimer’s disease, resulting in increased FAAH protein activity [25]. Interestingly, patients with the most severe cognitive impairment were found to have lowest levels of methylation. The *FAAH* gene promotor is also known to possess an estrogen-responsive element resulting in estradiol-dependent transcriptional activation (via demethylation) of FAAH [18]. Moreover, many studies have shown bidirectional interactions between the endocannabinoid and monoamine systems. For instance, *FAAH* gene knockout in mice has been found to increase serotonergic tone 4-fold in the frontal cortex [26]. In turn, Best and Regehr showed that activation of 5-HT2-type receptors by serotonin is able to evoke endocannabinoid release [27]. CB1 stimulation has also been shown to increase the firing activity of dopaminergic neurons and increase synaptic dopamine concentration [28]. Increased dopamine D3 receptor mRNA was observed in the brains of *FAAH* knock-in C385A mice; that is, lower FAAH function was associated with a greater number of dopamine D3 receptors in the brain [29]. These findings are relevant insofar as they demonstrate the functional overlap of the endocannabinoid and monoamine systems, which is an important consideration for developing new pharmacotherapeutics for MDD.

Genetic deletion of *FAAH* in mice results in 15-fold higher brain levels of AEA [30]. FAAH is widely distributed in the central nervous system (CNS), most prominently in the cerebral cortex, hippocampal formation, amygdala and cerebellum, and its regional distribution is correlated with the distribution of CB1 receptors as well as with the highest rates of AEA degradation [13,31,32,33]. CB1 receptors are also widely distributed in the CNS (particularly within the cerebral cortex, hippocampus, amygdala, striatum and substantia nigra) and have functional roles in mood, anxiety, pain, motor regulation, appetite control and memory processing [5,34,35]. Importantly, the ECS possesses plasticity; that is, the ECS is modulated in response to both acute and chronic stimuli. For instance, chronic exposure to exogenous agonists such as Δ^9^-THC results in reduced CB1 receptor density and signaling [36].

Alterations of the ECS are implicated in several neuropsychiatric disorders, such as Parkinson’s disease, Alzheimer’s disease, anxiety and MDD [37]. Human and animal data have shown that the ECS is involved in emotion regulation and cognition [38,39]. In response to stressors, the breakdown of AEA by FAAH is initiated, reducing AEA-CB1 signaling [34]. Moreover, corticotropin-releasing hormone, a stress-responsive neuropeptide, increases FAAH activity and thereby reduces AEA concentrations [40]. Studies linking stress and ECS dysregulation are of particular interest, since it is known that exposure to stressors can increase the risk of psychological disorders, including MDD [41]. Given the role of ECS signaling in mood, anxiety and cognition, it is likely that the ECS is hypofunctional (e.g., reduced AEA-CB1 signaling) in MDD and that increasing ECS signaling could elicit antidepressant-like effects. For example, the CB1 receptor antagonist, rimonabant, has been associated with an increased risk of developing or exacerbating depressive symptoms in humans, and genetic deletion of CB1 receptors in mice elicits a depressive-like phenotype [42,43]. These findings have triggered an interest in ECS-altering drugs to restore AEA-CB1 signaling. One such class of agents are FAAH inhibitors, which work by inhibiting FAAH activity, consequently increasing AEA concentration and stimulating downstream CB1 receptor signaling.

FAAH inhibitors are of particular interest, because they lack the same side effects that exogenous CB1 receptor agonists elicit, such as hypomotility, hypothermia and catalepsy [44]. Moreover, FAAH inhibition does not display reinforcing effects [44]. However, that is not to say that FAAH inhibitors do not carry their own risk of side effects, such as presyncope, diarrhea, and headaches [45]. Several irreversible FAAH inhibitors have been assessed in patients with MDD in clinical trials and, in fact, some were terminated due to adverse events. During a Phase I clinical trial of BIA 10-2474 conducted by Bial Pharmaceuticals, one participant died and five participants were hospitalized in January 2016 due to adverse effects of BIA 10-2474 [46]. It was speculated that BIA 10-2474 resulted in off-target effects that led to neurotoxicity [47]. Hence, there is a need to further investigate FAAH and develop safer, selective FAAH inhibitors. As previously described, first-line antidepressants are inadequate in alleviating depressive symptoms in approximately half of MDD patients, indicating a pressing need to develop alternative antidepressant pharmacotherapies. The aim of this scoping review was to evaluate the role of brain FAAH in depression in animal models and behavioral tests of depression, such as the chronic mild stress and forced swim test paradigms, to determine the therapeutic value of targeting FAAH in clinical trials.

## 2. Results

**Figure 2:**
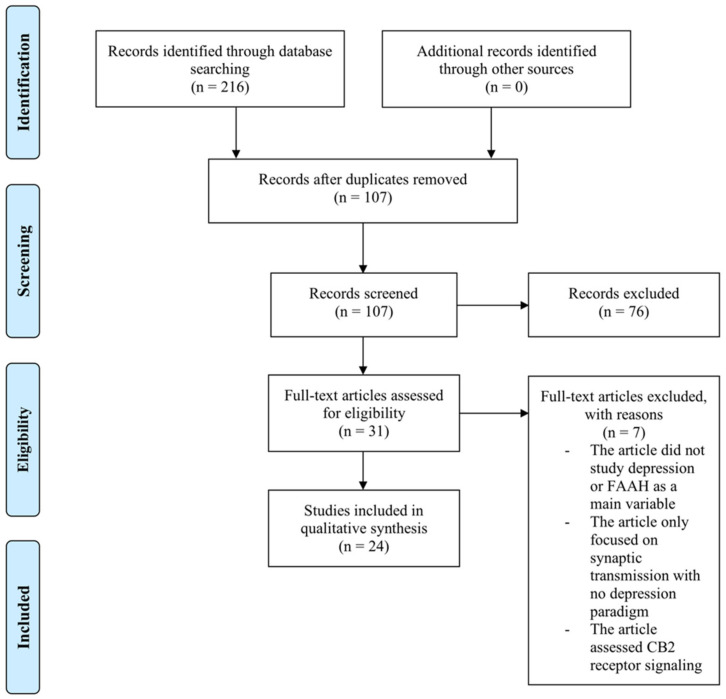
Preferred reporting items for systematic reviews and meta-analyses (PRISMA) flowchart of literature search results.

The literature search yielded 216 articles that were screened for inclusion on the basis of their titles, abstracts and full text (see Figure 2). After the removal of duplicates and ineligible articles, a final total of 24 articles were eligible and included in this review. The details of the included studies are described in Table 1.

### 2.1. Brain FAAH Gene Expression and Protein Levels in Animal Models of Depression

Many researchers have characterized the expression pattern of the *FAAH* gene and its corresponding protein levels in the brain. Given that stress upregulates FAAH activity and MDD patients experience greater stress levels, it is expected that *FAAH* gene expression (e.g., the mRNA product) and, consequently, FAAH protein levels or activity (e.g., the protein product) would also be upregulated in the depressive phenotypes [48]. Reich et al. tested the effects of chronic mild stress (CMS) on male and female rats [49]. Western blot analysis revealed significantly increased FAAH protein levels in the hippocampus of rats exposed to CMS compared to control rats. There were no sex differences in FAAH protein levels in either the rats exposed to CMS or control rats. The maternal deprivation model of depression has also been used to assess *FAAH* gene expression in male and female rats [50]. Gene expression was analyzed using real-time quantitative polymerase chain reaction (qPCR), which showed increased *FAAH* gene expression in the frontal cortex in males, whereas in females, increased *FAAH* gene expression was observed in the hippocampus. The frontal cortex is important for many cognitive functions, such as attention, emotional processing and affective control [9]. The hippocampus is also an important region, as it is vital for learning, memory and emotion, since it is functionally connected to other limbic brain regions [8]. These data reveal that the ECS is vulnerable to stress and that there may be altered brain *FAAH* gene expression and protein levels in depressive phenotypes. There may also be potential sex-dependent differences in FAAH, which may translate to differential responses to chronic stress and distinct depressive phenotypes between males and females.

Smaga et al. used Wistar–Kyoto (WKY) and olfactory bulbectomized (OBX) male rats, both of which are animal models of depression (the former being a genetic model), to assess FAAH protein levels in the brain [51]. They found increased FAAH protein levels in the PFC and hippocampus of OBX rats, accompanied with a decrease in AEA. In the WKY rats, increased FAAH protein levels were observed in the dorsal striatum and decreased FAAH protein levels in the nucleus accumbens. These changes in the ECS were paralleled by increased immobility time on the FST, which reflects greater behavioral despair. The observation that FAAH protein levels are altered in the dorsal striatum and nucleus accumbens is relevant as it suggests a mechanism that might exist where altered FAAH activity in mesolimbic areas influences dopaminergic neurotransmission [52]. This is important because dopaminergic signaling is vital for the reward circuits, directly relating to anhedonia in MDD [53]. A recent study by Huang et al. employed the chronic unpredictable stress (CUS) model to assess FAAH mRNA and protein levels in male mice [54]. They found that, after CUS, FAAH mRNA and protein levels were significantly increased in the mouse hippocampus. They also observed decreased N^6^-methyladenosine methylation (a posttranscriptional internal mRNA modification) of FAAH mRNA in the mouse hippocampus after CUS. Injection of circSTAG1 (also known as circRNA-mm9_circ_0015149) lentivirus into the mice resulted in overexpression of circSTAG1 and increased N^6^-methyladenosine methylation of FAAH mRNA and degradation of FAAH protein. This change was associated with attenuated depressive-like behaviors as measured by the forced swim, tail suspension and sucrose preference tests. Since decreased methylation of FAAH mRNA in the hippocampus was observed in CUS mice, this observation suggests that increased FAAH protein activity in the hippocampus might play a role in the manifestation of depressive symptoms, such as problems with memory. The hippocampus is structurally and functionally related to the PFC and other limbic regions (e.g., amygdala), which also makes it a highly attractive target for depressive symptomatology [8].

In contrast to the above studies showing increased *FAAH* gene expression and protein levels, one study found opposing results. Kirkedal et al. studied a genetic rat model of depression, the Flinders Sensitive Line, and their controls, the Flinders Resistant Line, using qPCR and Western blot analysis [55]. Interestingly, *FAAH* mRNA and protein levels were decreased in the right PFC and left hippocampus, respectively, of Flinders Sensitive Line rats. Another study of CUS failed to find any significant differences in FAAH [7]. CUS was applied to male rats, which resulted in a reduction in sexual motivation, consistent with what is observed in human patients with MDD. CUS also produced a reduction in AEA in the PFC, hippocampus, hypothalamus, amygdala, midbrain, and ventral striatum; intuitively, this would suggest higher FAAH enzymatic activity; however, there was no effect of CUS on FAAH enzymatic activity, which was measured using the conversion of [^3^H]-anandamide to [^3^H]-ethanolamine. Imipramine, a tricyclic antidepressant (TCA) that blocks serotonin and norepinephrine transporters as well as muscarinic acetylcholine receptors, was also administered. Imipramine had no significant effect on FAAH enzymatic activity when rats were unstressed. Interestingly, however, there was an interaction effect between imipramine and CUS, where rats who received both imipramine and CUS were observed to have increased FAAH enzymatic activity in the midbrain and ventral striatum, which was an unexpected effect. The authors suggested that the imipramine-induced increase in FAAH activity in the presence of CUS could be an undesirable side effect of imipramine treatment that may be opposing its typical antidepressant properties. There are other classes of antidepressants, besides TCAs, which have not been investigated with respect to *FAAH* gene expression or protein activity in animal models of depression. Therefore, it would be worthwhile to investigate other classes of antidepressants, such as SSRIs, to determine the effects on *FAAH* gene expression and protein activity, since SSRIs only block serotonin reuptake. Furthermore, there have only been two studies of an animal model of depression where a FAAH inhibitor is administered concomitantly with an antidepressant: one being a TCA (e.g., imipramine) and the other being an SSRI (e.g., fluoxetine) [56,57].

In summary, the majority of the studies that assayed *FAAH* gene expression and protein levels indicate that *FAAH* gene expression and protein levels are increased when the animal displays depressive-like symptoms, particularly within the PFC, hippocampus and striatum. These observations are paralleled by increased behavioral despair, which suggests a positive relationship between *FAAH* gene expression and protein levels and depressive-like behavior. Increased FAAH protein levels in the aforementioned brain regions would result in decreased AEA concentration and CB1 receptor signaling. Such dysregulation of ECS components in the PFC and hippocampus would result in deficits in attention, information processing, decision making and autobiographical memory, contributing to depressive symptoms in MDD [53]. Likewise, dysregulation of ECS components in the striatum could contribute to depressive symptoms, such as anhedonia due to disruption to the reward system [53].

### 2.2. Genetic Deletion of FAAH and Depressive-Like Behaviors

FAAH enzymatic activity can be altered through genetic manipulation; for example, its enzymatic activity can be diminished through its genetic deletion. Genetic deletion of the *FAAH* gene eradicates FAAH enzymatic activity, which raises AEA concentration in synapses. This allows for greater AEA-CB1 receptor interaction and downstream signaling that could, in turn, improve depressive severity. Naidu et al. investigated the effect of genetic deletion of *FAAH* as well as administration of URB597, a selective FAAH inhibitor, in mice [58]. At first, *FAAH* deletion and URB597 treatment (even across a wide dose range: 0.3, 1, 3, 10 mg/kg) failed to produce any significant effects on immobility time in the tail suspension test (TST). After making methodological changes (e.g., increasing sample size and altering ambient lighting), the authors found that *FAAH* knockout and URB597-treated (0.1 mg/kg) mice displayed significant reductions in immobility time in the modified TST. They concluded that pharmacological inhibition or genetic deletion of *FAAH* is ineffective in standard mouse models of emotional reactivity, suggesting that the effectiveness of FAAH inhibition is dependent on the levels of stress associated with the environmental conditions. Another group also investigated the effect of genetic deletion of *FAAH* on depressive-like behavior in male mice [59]. *FAAH* knockout mice were tested using the forced swim test (FST) and TST and, in contrast to Naidu et al.’s initial findings, the mice displayed reduced immobility time on both tests. The authors concluded that genetic deletion of *FAAH* was associated with antidepressant-like effects. Although genetic knockout studies of *FAAH* are limited and the findings are mixed, these studies lend evidence to support the observation that the inhibition of FAAH enzymatic activity might be an effective method to reduce depressive-like behaviors, under optimal environmental conditions.

### 2.3. Antidepressant-Like Effects of URB597, URB694 and Other FAAH Inhibitors

There are numerous studies testing the effects of various FAAH-inhibiting agents on depressive-like behavior in rodent models. By inhibiting FAAH, AEA concentration in the brain can be increased, which allows for greater AEA-CB1 signaling. The most common FAAH inhibitor used in preclinical studies is URB597, an irreversible FAAH inhibitor. Gobbi et al. investigated the effects of URB597 in male rat and mouse models of depression [60]. The data showed that URB597 (0.1 mg/kg, intraperitoneally (i.p.)) resulted in accumulation of AEA in the hippocampus and PFC in the rats. In the mouse TST, URB597 (0.03–0.3 mg/kg) injection significantly decreased immobility time in a dose-dependent manner. This effect was maximal at 0.1 mg/kg. In the rat FST, URB597 (0.1 mg/kg) injection significantly decreased floating time. The results from the TST and FST indicate an antidepressant-like effect of URB597. Importantly, conditioned place preference and drug-discrimination tests demonstrated that URB597 does not produce rewarding effects or mimic the effects of Δ^9^-THC, unlike direct-acting CB1 receptor agonists, meaning that FAAH inhibitors lack the potential to be addictive or psychoactive.

The CMS model has also been used in conjunction with URB597 treatment in male rats [61]. Chronic URB597 (0.3 mg/kg, i.p.) administration restored body weight and sucrose consumption, exhibiting antidepressant-like effects. Chronic URB597 treatment also increased AEA levels in the midbrain, striatum and thalamus, but not in the PFC and hippocampus; this is in contrast to Gobbi et al.’s study, which revealed that acute (as opposed to chronic) injection with the same FAAH inhibitor, URB597, resulted in AEA accumulation in the PFC and hippocampus. The authors speculated that this discrepancy may be due to prolonged exposure of FAAH-inhibiting agents downregulating AEA mobilization in select brain regions, such as the PFC and hippocampus. The authors also assayed FAAH enzymatic activity using [^3^H]-anandamide as a substrate, which showed that URB597 effectively produced inhibition of FAAH activity in the midbrain, striatum and hippocampus. Similarly, Jankovic et al. studied male and female rats exposed to CUS [62]. The authors found that CUS increased depressive-like behaviors as measured by increased immobility time on the FST in both males and females. The rats were also administered URB597 (0.3 mg/kg, i.p.), which resulted in reduced immobility time on the FST in both male and female rats.

Adamczyk et al. found that URB597 reduced the immobility time of male rats in the FST, indicating that indirect CB1 receptor activation, by way of FAAH inhibition, has antidepressant-like effects [56]. It should be noted that the reduction in immobility time was dose dependent, whereby 0.1 and 0.3 mg/kg, but not 0.03 mg/kg, were effective in reducing immobility time. URB597 was additionally administered concomitantly with imipramine, which also significantly reduced immobility time. This is of clinical relevance since concomitant administration of FAAH inhibitor plus antidepressant drugs may be more beneficial than either alone. For example, Hill et al. failed to show any significant effect of imipramine on FAAH enzymatic activity, whereas Adamczyk and colleagues demonstrated that URB597 in addition to imipramine was effective in reducing depressive-like behavior [7]. Another group replicated these results in male mice and found that URB597 (0.05–10 μg/mouse) produced antidepressant-like effects on the FST, dose dependently [57]. For example, co-administration of the sub-effective dose of fluoxetine (2.5 mg/kg, i.p.), an SSRI, potentiated the effect of the sub-effective dose of URB597 (0.01 μg/mouse, intracerebroventricularly (i.c.v)). The authors concluded that the antidepressant-like effects of URB597 are comparable to fluoxetine when administered alone at the effective dose or co-administered at sub-effective doses. These results are also supported by a study that found that FAAH inhibition by URB597 (0.3 mg/kg, i.p.) significantly increased sucrose consumption and decreased immobility time in the FST, in WKY rats [63]. Similar results have been discerned in the tail-pinch test of coping after URB597 administration. Haller et al. utilized URB597 to test its effects on coping styles in male rats and found that URB597 (0.1 and 0.3 mg/kg) led the rats to use more active coping styles (e.g., trials of removing the clamp from tail) [64]. Thus, the authors proposed that enhancement of ECS signaling may be beneficial in disorders such as MDD that are characterized by passive coping. One recent investigation used the maternal deprivation model of early life stress to induce depression in male and female rats [65]. The rats were then administered URB597 (0.4 mg/kg, i.p.). This group also found that FAAH inhibition by URB597 prevented depressive-like behavior and impairment in social behavior, as measured by the FST and social preference and recognition tests, respectively, in both male and female rats.

McLaughlin et al. administered URB597 (0.5 and 1 μg) microinjections to the dentate gyrus of the dorsal hippocampus in male rats [66]. The hippocampus was chosen as the site for microinjection, because it is a critical brain region implicated in MDD: the hippocampus displays neuroplasticity, is involved in learning and memory, and is sensitive to stress and glucocorticoids [66]. Moreover, the dentate gyrus exhibits the most CB1 receptor binding in the hippocampus [66]. In contrast to the previously mentioned studies, the results showed no significant effect of the microinjections on immobility time on the FST. It is possible that the failure of URB597 to reduce depressive-like behavior was due to insufficient doses to elicit a response or that local inhibition in the dentate gyrus is insufficient and more widespread inhibition is needed.

URB694 is another selective FAAH inhibitor that is analogous to URB597. Carnevali et al. examined URB694 in a rodent model of social stress-induced depression [67]. Male rats were exposed to repeated social stress based on a classical resident–intruder paradigm, which reduced body weight and sucrose preference as well as increased immobility time on the FST. The rats then received URB694 (0.1 mg/kg, i.p.), which increased central and peripheral AEA levels and normalized changes in body weight, sucrose preference and immobility time on the FST. The same group then replicated this experiment in female rats [68]. Rats were exposed to social isolation, which caused less weight gain, reduction in sucrose preference and increased immobility time in the FST. Inhibition of FAAH by URB694 (0.3 mg/kg, i.p.) significantly restored behavioral and physical effects. Thus, the authors posited that pharmacological enhancement of AEA, by way of inhibiting FAAH, could be a promising antidepressant strategy for both males and females.

In addition to binding to CB1 receptors, AEA also binds to transient receptor potential vanilloid type-1 (TRPV1) receptors. Once bound, TRPV1 exerts the opposite behavioral effects of CB1 (e.g., there is exacerbation of depressive-like behaviors). Kirkedal et al. investigated whether local injection of the FAAH/TRPV1 dual blocker, N-arachidonoylserotonin (AA-5HT), into the medial PFC of male rats would produce antidepressant-like effects [69]. The rats were then placed under the FST. They found that moderate doses of AA-5HT (0.250 nmol/0.4 μL/side) significantly reduced immobility time in the FST compared to vehicle. Lower (0.125 nmol/0.4 μL) and higher (0.5 nmol/0.4 μL) doses had no significant effect, demonstrating a dose-dependent effect that did not appear to be linear, but rather bell shaped. These results highlight the role of the ECS in the PFC in emotional behavior. Another group also investigated the ability of AA-5HT to reverse behavioral despair following stress in male rats [70]. Stress was induced by restraint and the FST was used to measure changes in behavioral despair. The data revealed that three injections of AA-5HT (5 mg/kg) reversed behavioral despair in the FST in the stressed rats (as indicated by changes in immobility, swimming and climbing) which supports Kirkedal et al.’s findings [69]. URB597 (0.3 mg/kg) was also administered, which produced similar effects in the rats under basal and stressed conditions. Therefore, AA-5HT appears to be effective in reversing behavioral despair (e.g., producing antidepressant-like effects in stressed rats).

Less common FAAH inhibitors, such as JZL195, PF3845 and SSR411298, have also been used in animal models of depression. JZL195 is a dual inhibitor that targets both FAAH and MAGL. The relationship between MAGL and depression has not been as well studied; however, some data suggest that dysregulated 2-AG signaling is implicated in both human and animal studies of depression and that inhibition of MAGL elicits antidepressant-like effects in mice [71,72,73]. A recent investigation used JZL195 to inhibit FAAH and MAGL in female rats to test the effect on depressive behavior [74]. The authors found that JZL195 (3 mg/kg, i.p.) increased sucrose consumption and social interaction in the rats, accompanied by a reduction in immobility time during the FST. Increased sucrose consumption is likely a result of restored ECS function and reward systems in the striatum. Therefore, dual inhibition of FAAH and MAGL also appears to be effective in producing antidepressant-like effects. Another irreversible FAAH inhibitor is PF3845, which has also been used to test for antidepressant-like effects [75]. To establish a model of depression, mice were administered either corticosterone or vehicle followed by PF3845. Depressive-like behavior was measured by the FST and sucrose preference test. They found that 10 or 20 mg/kg i.p. acute injections of PF3845, but not 5 mg/kg, and three daily injections of 10 mg/kg i.p. of PF3845 significantly reduced immobility time in mice that only received vehicle, but not in corticosterone-exposed mice. Finally, Griebel et al. tested the effects of the selective, reversible FAAH inhibitor, SSR411298, on depressive-like behavior in rats and mice [35]. SSR411298 at 1 and 3 mg/kg (i.p. or i.o.) produced antidepressant-like activity in the rat FST, indicated by reduced immobility time. SSR411298 at 10 mg/kg improved the physical state of the mouse coat. The physical state of the coat of the animal is used to assess the animal’s grooming practices, which reflect hygiene and self-care practices that are sometimes neglected in humans with MDD [76].

In summary, there is a large consensus across studies that inhibition of FAAH, using a range of inhibitors, is effective for producing antidepressant-like effects and restoring a normal phenotype on various behavioral tests of depression. The most common FAAH inhibitor used in preclinical studies is URB597, which demonstrates considerable antidepressant-like activity. Dual inhibitors, such as JZL195 and AA-5HT, and FAAH inhibitors in conjunction with an antidepressant treatment, also seem to be effective in reducing depressive-like behavior in animals. The brain regions that are most influenced by FAAH inhibitors are the PFC, hippocampus, and striatum; dysregulated FAAH activity in these regions could explain MDD symptoms such as loss of motivation, cognitive dysfunction, and anhedonia [53]. Moreover, preclinical studies of FAAH inhibition also reveal some sex differences in depressive phenotypes; still, FAAH inhibitors seem to effectively reduce depressive-like behaviors in both male and female animals [62,65,68]. A number of studies provided evidence of dose-response effects that must be further studied and will be relevant for new clinical trials [56,57,60,69,75].

Organizing the studies by animal model, several studies showed that CMS/CUS is associated with greater *FAAH* gene expression and protein levels and that FAAH inhibition effectively reduces depressive behavior. Maternal deprivation and WKY studies also revealed greater *FAAH* gene expression and protein levels and that FAAH inhibition effectively reduces depressive behavior. FSL rats showed decreased *FAAH* mRNA and protein levels, in contrast to other animal models. In healthy strains, there is generally greater *FAAH* gene expression and protein levels. In the resident–intruder paradigm, social isolation, corticosterone injection, and restraint models, as well as in healthy strains, FAAH inhibitors typically elicited antidepressant-like effects.

## 3. Discussion

This review aimed to establish the role of FAAH in animal models of depression to determine the therapeutic value of targeting FAAH in clinical studies. From the review of the literature, several key findings have emerged.

First, of the studies assaying *FAAH* gene expression and protein levels, the majority (four out of six studies) of the literature showed increased *FAAH* gene expression and protein levels in the brain, particularly in the PFC, hippocamps, and striatum, when the animal manifested depressive symptomology. Furthermore, increased *FAAH* gene expression and protein levels are accompanied by increased depressive-like behavior as measured by tests such as the FST. As Huang et al. demonstrated, once FAAH is degraded, depressive-like behaviors are also attenuated [54]. Therefore, a positive relationship could be established between brain *FAAH* gene expression and protein levels and depressive-like behaviors. Greater *FAAH* gene expression and protein activity results in greater degradation of AEA and thereby reduced AEA-CB1 signaling. This relationship is consistent with studies of other ECS components. For example, it has been found that there is reduced AEA concentrations and increased CB1 receptors in rats displaying depressive symptomology [63]. It has also been observed that activation of CB1 receptors with exogenous agonists elicits antidepressant-like effects [77,78]. Therefore, it appears that normal AEA-CB1 signaling is important for healthy mood, and it can be indirectly regulated via FAAH. Interestingly, in contrast to the other studies, one study by Kirkedal and colleagues found the opposite relationship, where decreased *FAAH* mRNA and protein levels were observed in Flinders Sensitive Line rats [55]. A possible reason for the discrepant finding is that the authors took a hemispheric approach to analyzing ECS components, whereas other groups may have pooled data from both hemispheres. Pooling of data from both hemispheres would mask any differences in ECS components between each hemisphere. The discrepant finding could also be due to differences in the animal model and species strain used across studies; this study used Flinders Sensitive Line rats, a genetic model of depression, whereas another study assessing FAAH protein levels used Sprague–Dawley rats and a CMS model. Additionally, Hill et al. did not find any association between depressive-like state and FAAH enzymatic activity, but rather found that the TCA, imipramine, increased FAAH enzymatic activity only in the presence of CUS [7].

Second, 14 studies demonstrated that genetic knockout or pharmacological inhibition of FAAH elicited antidepressant-like effects, reduced depressive-like behavior, and restored normal behavioral phenotypes. An important distinction to make between genetic knockout of *FAAH* and pharmacological inhibition of FAAH is that, since knockout animals never produce the FAAH protein, the complete absence of FAAH may be different from animals that had normal FAAH function but were then treated with an inhibitor; thus, phenotypic outcomes may also be different. The data from the genetic knockout studies of *FAAH* are scarce and mixed. Of the two available genetic knockout studies, Naidu et al.’s investigation failed to find any initial significant effects of *FAAH* knockout or inhibition on depressive-like behavior, which is inconsistent with the results of the other studies [58]. However, once methodological changes were instituted (namely, increasing sample size and altering lighting conditions to resemble Gobbi et al.’s study [60]), Naidu and colleagues were able to find a significant reduction in depressive-like behaviors. The initial failure to find any significant effects could be attributed to a small sample size and an altered methodology. The authors also suggested that the efficacy of *FAAH* knockout or inhibition may be dependent on the stress levels associated with the environmental conditions. A number of studies reviewed here suggest that FAAH expression increases in response to early life stress or chronic stress. Other data also suggest that FAAH activity is increased in response to stressors and stress hormones [34,40]. It is possible, then, that the efficacy of *FAAH* knockout or inhibition is dependent on the severity of stress the animal experiences and the extent to which *FAAH* gene expression or protein level is increased. There is a large consensus across animal studies that FAAH inhibition is an effective method of reducing depressive-like behavior. This is a promising result, because it highlights the potential role of FAAH-inhibiting agents as alternative antidepressants. The most commonly used FAAH inhibitor was URB597, although other FAAH inhibitors (both reversible and irreversible) or dual inhibitors were also effective in reducing depressive-like behaviors. Only one study found no significant effects of FAAH inhibition by URB597; although, the authors employed microinjections of URB597 (0.5 and 1 μg) into the dentate gyrus of the hippocampus, whereas the other studies used larger doses by way of intraperitoneal injection [66]. Therefore, it is possible that the doses used in this study were not sufficient to inhibit FAAH activity and that a more widespread inhibition of FAAH activity in other brain regions in the neural circuitry of depression, such as the PFC, is required to rescue depressive phenotypes.

Notably, there appears to be dose-dependent effects with respect to the use of FAAH inhibitors. For example, Adamczyk and colleagues found that 0.1 and 0.3 mg/kg of URB597, but not 0.03 mg/kg, was sufficient in eliciting a behavioral response [56]. Similarly, Gobbi and colleagues found that 0.1 mg/kg, but not lower doses, was necessary for reducing depressive-like behaviors for both the FST and TST [60]. It is important to establish effective doses of each class of FAAH inhibitors for both future preclinical and clinical studies. For example, the effective doses for URB597 and URB694 evidently are 0.1–0.3 mg/kg in rodents. Determining how effective doses in rodents translate to salubrious effects in such a way that depressive symptoms are significantly attenuated will be an important avenue to pursue in human clinical research. Another valuable observation is that animals treated with FAAH inhibitors did not show Δ^9^-THC-like effects or deficits in locomotion, which are common side effects of direct-acting CB1 agonists. Furthermore, FAAH inhibitors do not elicit reinforcing or rewarding effects, indicating that FAAH inhibitors lack addictive properties [60].

Third, a large number of studies found significant differences in FAAH mainly in the PFC, hippocampus and, to a lesser extent, the striatum. These regions are not only highly implicated in depression [9], but they are also regions where greater density of ECS components are typically found [7]. Identifying the brain regions where ECS dysregulation is occurring is important in order to make connections with depressive symptoms. The PFC has a vital role in a myriad of cognitive functions including decision making, attention, motivation, problem solving as well as emotion and affect regulation [8,9,79]. Aberration in normal ECS signaling in the PFC could be related to functional impairments in MDD, such as inability to pay attention, lack of motivation, sad mood, or difficultly making daily decisions [8]. For example, Fagundo et al. showed that an increase in AEA levels is associated with improved decision making and cognitive flexibility in healthy female participants [80]. Therefore, the PFC is an important brain region of interest with respect to ECS dysregulation. In fact, the PFC is a prominent target for other treatment modalities, such as brain stimulation techniques, in MDD patients [81]. The hippocampus is also an important region in MDD as it is vital for learning, memory, and neuroplasticity [9]. The hippocampus is known to be affected in depression and is connected to emotion-related brain regions such as the PFC and amygdala [9]. Interestingly, greater connectivity between fronto-limbic regions has been associated with greater depressive severity [82]. It is widely accepted that patients with MDD have smaller hippocampal volumes and decreased hippocampal activity [83,84]. Moreover, stress can result in elevated glucocorticoids, which act on glucocorticoid receptors in the hippocampus leading to hippocampal atrophy [85]. Functionally, this could result in issues with autobiographical memory and negative emotion [53]. Other data suggest that CB1 receptor activation and endogenous release of AEA modulate memory consolidation [86,87]. Lastly, dysregulation of the ECS in the striatum could correlate with anhedonia in depression; in fact, a number of the studies reviewed here demonstrated this phenomenon using the sucrose preference test [88]. Disruption to dopaminergic signaling and decreased reward network connections have been associated with greater depression severity [89]. Functionally, these findings may translate to lack of response to rewarding or positive stimuli in MDD patients. Dysfunction in the ECS and aberrations in normal CB1 receptor signaling in these key brain regions, combined with crosstalk with other neurotransmitter systems, could play a significant role in depressive symptomology. Identifying the brain regions where ECS dysfunction has the most profound impact can help identify brain–behavior relationships with respect to specific symptoms of MDD. Therefore, the PFC, hippocampus and striatum should be considered treatment targets in clinical studies and key brain regions of interest in future neuroimaging studies in MDD patients.

Lastly, a number of studies assessed sex differences with respect to FAAH. While two studies found significant sex differences in FAAH, three studies found no differences in FAAH between the sexes [49,50,62,65,67,68]. Evidently, the data on sex differences in FAAH are mixed, which could be due to differences in the animal model or species strain. Additionally, the majority of studies on FAAH in animal models of depression used male mice and rats; this is the case in most animal studies largely due to the assumption that female estrous cycles may confound the effects of experimental manipulations [90]. However, there is a nearly 2-fold greater prevalence of MDD in women compared to men [91]. Therefore, future studies should employ female animals in preclinical studies to allow for the generalization of results involving male animal studies and to further investigate sex differences in the ECS, particularly FAAH expression. Uncovering such differences will be important to consider when targeting FAAH in clinical trials, since it is known that there are sex differences in response to current antidepressant treatments [92].

FAAH is a promising therapeutic target for many disorders of the CNS. Numerous potent and selective FAAH inhibitors have been developed for clinical trials in both healthy and patient populations. To our knowledge, there have only been two clinical trials evaluating FAAH inhibitors in MDD to date. One is a Phase II trial (NCT00822744, 2008-001718-26) that evaluated the efficacy of SSR411298 in elderly patients with MDD. SSR411298 did not show any significant improvement in depressive severity as measured by the Hamilton Depression Rating Scale [93]. Although at first glance this result seems unfavorable, FAAH inhibition could be potentially more effective in a younger population, since MDD differs considerably between younger and older adults [94]. The second clinical trial (NCT02498392, 2015_002007_29) evaluated the efficacy of JNJ-42165279 in participants with MDD with Anxious Distress. No results have been published to date. Therefore, more clinical trials of safe and effective FAAH inhibitors are needed in MDD.

### 3.1. Limitations

The main limitation of modeling depression in animals is that pure human features of depression, such as guilt, sad mood, and suicidality, are difficult to model in animals [95]. Currently, symptoms such as despair, anhedonia, helplessness and changes in weight or appetite can be assessed in animal models of depression. Even though specific behavioral tests have been developed for depression (e.g., FST) and anxiety (e.g., elevated plus maze), inducing stress produces both depressive-like and anxiety-like effects in animals, which makes it difficult to distinguish between depressive and anxious behavior. In this review, we excluded publications assessing anxiety-like behavior in order to assess the function of FAAH in isolated cases of depression. It is possible, then, that the publications included in this review found differences in FAAH related to anxiety-like behavior but not depressive-like behavior. Moreover, variations in animal models produce confounding variables across studies, making it difficult to arrive at the same conclusions or to compare across studies. One animal model (e.g., CMS) might find a different outcome compared to a study that employed a different animal model (e.g., genetic model), despite controlling for strain.

### 3.2. Future Directions

An important avenue to pursue is the interaction between the monoaminergic and endocannabinoid systems as it will inform the best use of FAAH inhibitors, which may be used alone or in conjunction with other antidepressants. As previously mentioned, future studies should continue to explore sex differences in the ECS and focus on female samples in preclinical studies. Importantly, future studies may also consider incorporating in vivo imaging techniques to characterize the ECS in patients with MDD. This will be an important step in informing future clinical trials of FAAH inhibitors in MDD.

## 4. Materials and Methods

### 4.1. Search Strategy

A comprehensive search of the literature using PubMed, Scopus and Embase databases was performed (all hits until October 2020) according to the PRISMA statement [96]. The search terms consisted of “fatty acid amide hydrolase or FAAH and depressi*”.

### 4.2. Selection Criteria

Only peer-reviewed primary journal articles assessing fatty acid amide hydrolase in animal models of depression were included in this review. The language of publication was limited to English. Both longitudinal and cross-sectional studies were included. Publications that studied any of the following as the main variable were excluded: (1) pain, (2) alcohol use, (3) nicotine use, (4) posttraumatic stress disorder, (5) anxiety, (6) CB2 receptor expression, and (7) synaptic transmission.

### 4.3. Data Extraction

The following data were extracted from each eligible study: (1) the year the study was published; (2) species (e.g., mice or rats); (3) sex; (4) model of depression (e.g., maternal deprivation or chronic mild stress); (5) measure of FAAH (e.g., Western blot analysis or FAAH inhibitor administration); (6) dose of FAAH inhibitor, if applicable; and (7) measure of depressive symptoms (e.g., forced swim test or sucrose consumption test).

### 4.4. Common Animal Behavioral Tests of Depression

Forced swim test. In the forced swim test, the rodent is placed in a glass cylinder of water and, after a period of struggling and escape behavior, the rodent eventually becomes immobile [97]. The time spent immobile is measured as an index of behavioral despair, reflecting the depressive phenotype.

Tail suspension test. The tail suspension test is a behavioral test of depression, where the rodent is suspended from its tail [98]. During this time, the rodent will display struggling and escape behaviors. Similar to the FST, the immobility time is measured as an index of behavioral despair.

Sucrose preference test. The sucrose preference test is used as a measure of sensitivity to reward [99]. The rodent is given water with and without different concentrations of sucrose and the preference rate for sucrose is measured. Since one symptom of MDD is loss of interest or pleasure, the preference rate for sucrose is used as an index of anhedonia, where reduced preference for sucrose is thought to be representative of anhedonia.

Social interaction test. The social interaction test consists of three rooms or chambers: social, neutral and anti-social [74]. A novel rodent is placed into the social chamber before introducing the subject rodent. The time spent in each chamber and any social interactions (e.g., sniffing, touching) are recorded. Spending more time in the anti-social chamber is reflective of the social withdrawal symptom of MDD.

Tail-pinch test of coping. In the tail-pinch test, a clamp is attached to the tail of the rodent [100]. Attaching the clamp to the rodent’s tail is a mildly stressful event; naturally, the rodent will attempt to escape. Trials of removing the clamp are recorded as active responses, whereas ignoring the clamp is recorded as a passive response. Passive responses, or poor coping, are reflective of depressive phenotypes, namely avoidant behavior [65].

### 4.5. Common Animal Models of Depression

Resident–intruder paradigm. The resident–intruder paradigm is used to induce social stress and subsequent depressive behaviors in rodents [101]. In this paradigm, the intruder rodent is transferred to the resident rodent’s cage, usually resulting in investigation, domination, and defeat of the intruder rodent by the resident rodent, due to dominance hierarchies that exist within male rodents [102]. The intruder rodent experiences consequent behavioral changes, such as reduced social interaction. In humans experiencing social defeat, there is also a greater severity of depression, loneliness, and social withdrawal. Social stress is also associated with changes to the serotonergic and noradrenergic systems in animals [103].

Chronic mild (or unpredictable) stress. Chronic mild stress, also known as chronic unpredictable stress, is a model of inducing a chronic depressive-like state and involves the application of physical stresses (e.g., food or water deprivation, changes to climate) intermittently over a prolonged period of time. Depressive symptomology results, such as anhedonia and decreased interest in sexual and self-care behaviors, which are paralleled by neurochemical changes in the dopaminergic system [104].

Maternal deprivation. Maternal deprivation is a model of early life stress, where a pup is separated from its mother for 1–24 h in the early postnatal period, which is a critical period for establishing neural circuits regulating behavior [105]. As adults, animals exposed to maternal deprivation show increased hypothalamic–pituitary–adrenal (HPA) response and display depressive-like behaviors [106]. In humans, early adverse events such as inadequate parental care is associated with greater risk of both physical and mental illnesses [106].

Olfactory bulbectomy. Olfactory bulbectomy is another method used to induce depressive-like symptoms in animals. Since the rat olfactory bulb forms a part of the limbic system, when the animal is surgically bulbectomized, the outcome involves neuronal degeneration of the cortical–hippocampal–amygdala circuit, circuitry which is also dysfunctional in MDD patients. These changes are associated with reduced concentrations of brain norepinephrine and serotonin, which manifests as depressive-like behaviors resembling those of MDD patients [107].

Social isolation. Social isolation is used to induce depression in animals, mimicking the effects of isolation in social animals, such as humans, which is known to have detrimental effects for both physical and mental health [108]. Social isolation acts as a stressor, resulting in depressive-like behavior and increased HPA activation [109,110].

Genetic models of depression. Wistar–Kyoto and Flinders Sensitive Line rats are both genetic models of depression. These rats are selectively bred to produce genetically vulnerable strains with increased learned helplessness behavior [111,112].

Corticosterone injection. The corticosterone animal model of depression consists of repeated corticosterone injections, which increase depressive-like behavior. It serves as a stress-induced depression model [113].

## 5. Conclusions

In conclusion, the current animal models of depression show that *FAAH* gene expression and protein levels are dysregulated in the brain; specifically, there is increased *FAAH* gene expression and protein activity in the PFC, hippocampus and striatum, which correlates with the depressive phenotype. Moreover, FAAH-inhibiting agents attenuate depressive symptoms, without the side effects of direct-acting CB1 agonists. Even though results of the limited clinical trials of FAAH inhibitors in MDD have been unfavorable or unknown, the preclinical data suggest that FAAH is a promising therapeutic target for pharmacological intervention in humans with MDD and provides opportunities for combining FAAH inhibitors with other treatment modalities, such as monoamine-elevating antidepressants, transcranial magnetic stimulation, or electroconvulsive shock therapy. Therefore, more research should be performed using neuroimaging techniques in order to identify neural circuits or neurochemistry related to FAAH in vivo, and alternative FAAH inhibitors in clinical trials.

## Figures and Tables

**Table 1:** Overview of the eligible studies included in this review.

Study	Species	Sex	Model of Depression	Measure of FAAH	Dose of FAAH Inhibitor	Measure of Depressive Symptoms
Hill et al. 2008	Long Evans rats	Male	Chronic unpredictable stress	[3H]-anandamide conversion to [3H]-ethanolamine	N/A	Sexual motivation
Reich et al. 2009	Sprague-Dawley rats	Males and females	Chronic mild stress	Western blot analysis	N/A	N/A
Marco et al. 2014	Albino Wistar rats	Males and females	Maternal deprivation	Quantitative polymerase chain reaction	N/A	N/A
Smaga et al. 2017	Wistar and Wistar Kyoto rats	Male	Wistar Kyoto and bulbectomized rats	Western blot analysis	N/A	Forced swim test
Kirkedal et al. 2019	Flinders line rats	Male	Flinders Sensitive Line	Quantitative polymerase chain reaction and Western blot analysis	N/A	N/A
Huang et al. 2020	C57BL/6J mice	Male	Chronic unpredictable stress	Polymerase chain reaction and Western blot analysis	N/A	Forced swim test, sucrose preference test, tail suspension test
Naidu et al. 2007	ICR, C57BL/6 and *FAAH* (-/-) mice	Unknown	Healthy	*FAAH* gene knockout; URB597 administration	0.3, 1, 3, or 10 mg/kg	Forced swim and tail suspension tests
Bambico et al. 2010	Mice	Male	Healthy	*FAAH* gene knockout	N/A	Forced swim test, tail suspension test
Gobbi et al. 2005	C57BL/6 mice, Wistar rats, Sprague-Dawley rats	Male	Healthy	URB597 administration	0.03–0.3 mg/kg	Forced swim and tail suspension tests, conditioned place preference test and drug discrimination test
McLaughlin et al. 2007	Sprague-Dawley rats	Male	Healthy	URB597 administration	0.5 and 1 μg	Forced swim test
Bortolato et al. 2007	Wistar rats	Male	Chronic mild stress	URB597 administration; [3H]-anandamide conversion to [3H]-ethanolamine	0.3 mg/kg, i.p.	Body weight, sucrose consumption
Adamczyk et al. 2008	Wistar rats	Male	Healthy	URB597 administration	0.03, 0.1 or 0.3 mg/kg	Forced swim test
Umathe et al. 2011	Swiss mice	Male	Healthy	URB597 administration	0.05–10 μg/mouse	Forced swim test
Vinod et al. 2012	Wistar Kyoto rats	Male	Wistar Kyoto rats	URB597 administration; immunoblot analysis	0.3 mg/kg, i.p.	Forced swim test, sucrose consumption
Haller et al. 2013	Wistar rats	Male	Healthy	URB597 administration	0.1 and 0.3 mg/kg	Clamp removal during tail pinch test
Alteba et al. 2020	Sprague-Dawley rats	Males and females	Maternal deprivation	URB597 administration	0.4 mg/kg, i.p.	Forced swim test, social preference test, social recognition test
Jankovic et al. 2020	Wistar rats	Males and females	Chronic unpredictable stress	URB597 administration	0.3 mg/kg, i.p.	Forced swim test
Carnevali et al. 2015	Wistar Kyoto and wild-type Groningen rats	Male	Resident-Intruder paradigm	URB694 administration; FAAH assay	0.1 mg/kg, i.p.	Sucrose preference test, forced swim test, body weight
Carnevali et al. 2020	Wild-type Groningen rates	Female	Social isolation	URB694 administration; FAAH assay	0.3 mg/kg, i.p.	Sucrose preference test, forced swim test, body weight
Wang and Zhang 2017	C57BL/6 and CD1 mice	Male	Corticosterone administration	PF3845 administration	Acute injections of 5, 10 or 20 mg/kg, i.p. or three daily injections of 10 mg/kg i.p.	Forced swim test, sucrose preference test
Griebel et al. 2018	BALB/c, CD1, NMRI, OF1 and Swiss mice; Sprague-Dawley, Wistar and Long Evans rats	Male	Chronic mild stress	SSR411298 administration	0.3-30 mg/kg, i.p. or o.p.	Forced swim test, coat condition
Dong et al. 2020	Wistar Kyoto rats	Female	Wistar Kyoto rats	JZL195 administration	3 mg/kg, i.p.	Sucrose preference test, social interaction test, forced swim test
Navarria et al. 2014	Wistar rats	Male	Restraint	AA-5HT and URB597 administration, FAAH assay	AA-5HT: 2.5 and 5 mg/kg, i.p.; URB597: 0.3 mg/kg, i.p.	Forced swim test
Kirkedal et al. 2017	Sprague-Dawley rats	Male	Healthy	AA-5HT administration	0.125, 0.250 or 0.500 nmol/0.4 μL per cortical hemisphere	Forced swim test

Abbreviations: N/A, not applicable; FAAH, fatty acid amide hydrolase; i.p., intraperitoneally; p.o., orally.

## Abbreviations

2-AG	2-arachidonylglycerol
AA-5HT	N-arachidonoylserotonin
AC	Adenylyl cyclase
AEA	Anandamide
cAMP	Cyclic AMP
CB1	Cannabinoid 1
CB2	Cannabinoid 2
CMS	Chronic mild stress
CNS	Central nervous system
CUS	Chronic unpredictable stress
DAGL	Diacylglycerol lipase
ECS	Endocannabinoid system
FAAH	Fatty acid amide hydrolase
FST	Forced swim test
HPA	Hypothalamic–pituitary–adrenal
MAGL	Monoacylglycerol lipase
MAPK	Mitogen-activated protein kinase
MDD	Major depressive disorder
MDE	Major depressive episode
NAPE	N-arachidonoyl-phosphatidyl-ethanolamine
NAPE-PLD	N-acylphosphatidylethanolamine-selective phospholipase D
OBX	Olfactory bulbectomized
PFC	Prefrontal cortex
PKA	Protein kinase A
PRISMA	Preferred reporting items for systematic reviews and meta-analyses
qPCR	Quantitative polymerase chain reaction
SSRI	Selective serotonin reuptake inhibitor
TCA	Tricyclic antidepressant
Δ^9^-THC	Δ^9^-tetrahydrocannabinol
TRPV1	Transient receptor potential vanilloid type-1
TST	Tail suspension test
WKY	Wistar–Kyoto
